# Reelin Signaling in the Migration of Ventral Brain Stem and Spinal Cord Neurons

**DOI:** 10.3389/fncel.2016.00062

**Published:** 2016-03-11

**Authors:** Ankita R. Vaswani, Sandra Blaess

**Affiliations:** Neurodevelopmental Genetics, Institute of Reconstructive Neurobiology, Life and Brain Center, University of BonnBonn, Germany

**Keywords:** dopaminergic neurons, cranial motor neurons, somatic motor neurons, Dab1, preganglionic neurons, midbrain, hindbrain, mouse models

## Abstract

The extracellular matrix protein Reelin is an important orchestrator of neuronal migration during the development of the central nervous system. While its role and mechanism of action have been extensively studied and reviewed in the formation of dorsal laminar brain structures like the cerebral cortex, hippocampus, and cerebellum, its functions during the neuronal migration events that result in the nuclear organization of the ventral central nervous system are less well understood. In an attempt to delineate an underlying pattern of Reelin action in the formation of neuronal cell clusters, this review highlights the role of Reelin signaling in the migration of neuronal populations that originate in the ventral brain stem and the spinal cord.

## Introduction

The first indication for the role of Reelin in neuronal migration during brain development came from studies in *reeler* mice (Falconer, 1951; D’Arcangelo et al., 1995). In addition to a reeling, ataxic gait, to which these mutants owe their name, *reeler* mice exhibit improper cortical and hippocampal layering, cerebellar atrophy and ectopia of several neuronal populations (Falconer, 1951; Caviness and Sidman, 1973; Caviness, 1982; Goffinet, 1984b; Sheppard and Pearlman, 1997). Hence, this mutant strain was studied as a model for disrupted neuronal lamination long before the gene product responsible for its characteristic phenotype was identified, and it was established that defective neuronal migration is the primary cause of the *reeler* phenotype (Caviness and Sidman, 1973; Caviness, 1982). The discovery of the gene *Reelin*, as being the site of a deletion in *reeler* mice, paved way for its identification as an important regulator of neuronal migration and heightened interest in its exact role and mechanism of action (D’Arcangelo et al., 1995).

In what is described as the canonical signaling pathway, the product of the *Reelin* gene, a large extracellular matrix molecule, binds apolipoprotein E receptor 2 (ApoER2), also known as low-density lipoprotein receptor-related protein 8 (LRP8) or very low density lipoprotein receptor (VLDLR; Trommsdorff et al., 1999). This binding event results in the phosphorylation of the intracellular downstream effector disabled homolog 1 (Dab1) through the Src-family (rous sarcoma oncogene) tyrosine kinases Fyn (Fyn proto-oncogene) and Src (Howell et al., 1997; Hiesberger et al., 1999; Arnaud et al., 2003; Ballif et al., 2003). While downstream signaling events are not completely understood, the phosphorylated Dab1 molecule has been shown to be capable of recruiting several signaling pathways such as the Crk/CrkL-C3G-Rap1 pathway (Crk: adapter molecule crk; CrkL: Crk-like; C3G: Rap guanine nucleotide exchange factor 1; Rap1: Ras-proximate-1) to promote cell adhesion, or the LimK1-Cofilin1 pathway (LimK1: LIM domain kinase 1) that stabilizes the cytoskeleton (Park and Curran, 2008; Voss et al., 2008; Chai et al., 2009).

Most of the work on Reelin signaling and function has focused on its role during development of dorsal, laminar brain structures such as the cerebral cortex, hippocampus and cerebellum. From these studies, the following functions for Reelin signaling in neuronal migration emerge: stabilization of the leading process of migrating neurons, regulation of neuronal cell orientation or polarity, function as a stop signal for migrating neurons and indirect effects on neuronal migration e.g., by regulating the morphology and maturation of radial glia (for detailed recent reviews, refer to Sekine et al., 2004; D’Arcangelo, 2014; Förster, 2014).

Reelin also regulates radial and tangential migration of neurons that settle in the ventral brain stem and spinal cord; these cells are primarily organized into cell clusters. The underlying principles of Reelin function are less well understood in the development of these neuronal populations. Here, we will review the proposed roles of Reelin in the migration of neurons in the ventral brain stem and highlight the common themes in this regulation. We will focus in particular on the neuronal populations that are generated in the ventral progenitor domain of the brain stem and spinal cord. Nuclei derived from dorsal progenitor domains such as the pontine nucleus, dorsal cochlear nucleus, lateral reticular nucleus and inferior olivary complex will not be discussed.

## Ventral Midbrain

The ventral midbrain contains a number of nuclei derived from the ventricular zone in the ventral midbrain including the oculomotor nucleus, the red nucleus and midbrain dopaminergic (mDA) neurons. mDA neurons are arranged in three distinct anatomical clusters, the substantia nigra pars compacta (SNc), the ventral tegmental area (VTA) and the retrorubral field (RRF). The final position of the red nucleus and oculomotor neurons suggests that they undergo a one-step radial migration, while mDA neurons migrate first radially followed by a tangential migration step of SNc-mDA neurons (Prakash et al., 2009; Bodea et al., 2014).

### Expression Pattern of Reelin and Its Downstream Pathway Components in the Ventral Midbrain

**Figure 1:**
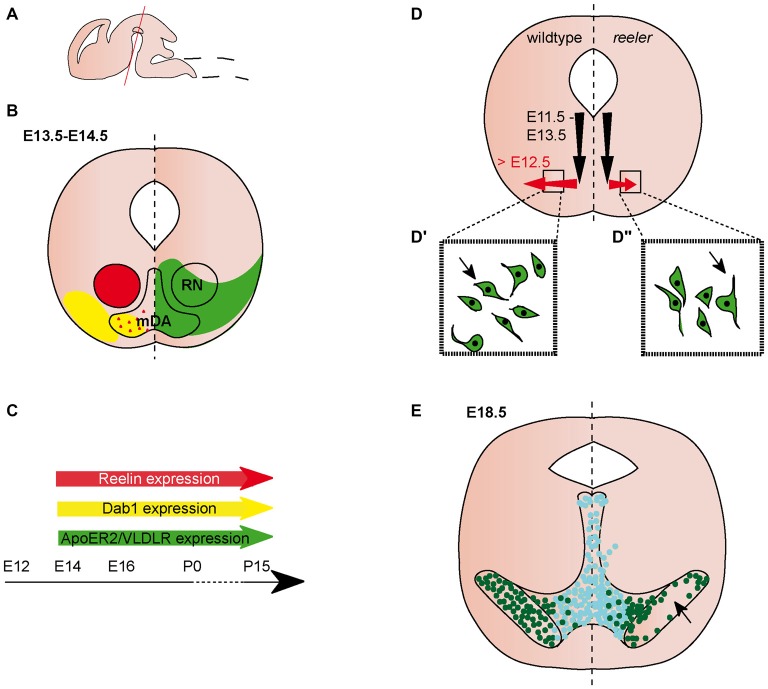
**Migration of midbrain dopaminergic (mDA) neurons. (A)** Sagittal view of embryonic brain. Red line indicates level of coronal sections in **(B,D,E)**. **(B)** Expression patterns of Reelin (red), Dab1 (yellow), ApoER2 (green) and very low density lipoprotein receptor (VLDLR; green) at the indicated time point. Red triangles in the mDA neuron region indicate Reelin secreted by striatal projections. Reelin and Dab1 expression are only presented in the left half of the brain, ApoER2 and VLDLR expression are only presented in the right half of the brain. Expression in the dorsal midbrain is not included in the schematic. **(C)** Expression of Reelin, Dab1, ApoER2 and VLDLR in the midbrain is maintained into adulthood. **(D)** Schematic of migratory paths of mDA neurons in wildtype (left half of the brain) and *reeler* mice (right half of the brain). Black arrows indicate radial migration, red arrows indicate tangential migration. Tangential migration is truncated in *reeler* mutants **(D^′^,D^″^**). In wildtype mice **(D^′^)**, processes of migrating mDA neurons are oriented tangentially, in *reeler* mice **(D^″^)** processes are oriented radially. **(E)** mDA neuron distribution in E18.5 wildtype mice (left half of the brain) and *reeler* mutants (right half of the brain). Turquoise dots represent Calbindin positive mDA neurons of the ventral tegmental area (VTA); dark green dots represent Girk2 positive mDA neurons of the substantia nigra (SN). SN-mDA neurons separate from VTA-mDA neurons but fail to reach their normal lateral location in *reeler* mice (arrow).

Both *Reelin* and *Dab1* expression are observed in the lateral ventral midbrain at E (embryonic day) 11.5 (Allen Institute for Brain Science, 2015). From E13.5 to early postnatal stages, *Reelin* expression is localized to the red nucleus (Bodea et al., 2014; Allen Institute for Brain Science, 2015; Figures 1A–C). After E15.5, its expression extends to additional regions in the ventral midbrain (Ikeda and Terashima, 1997; Allen Institute for Brain Science, 2015). *Reelin* mRNA is not expressed in mDA neurons at embryonic stages (Bodea et al., 2014), but there are conflicting reports on the presence of Reelin protein in the area where mDA neurons are located. Sharaf et al. (2014) report no immunoreactivity for Reelin (using a monoclonal antibody, clone G10; de Bergeyck et al., 1998) in mDA neurons at E16.5, P (postnatal day) 15 and P90. However, the authors found evidence for intra- and extracellular Reelin expression in mDA neurons at P0 (Sharaf et al., 2014). In contrast, immunostaining with the CR-50 antibody (Miyata et al., 1996), detects Reelin protein in the extracellular space surrounding mDA neurons (but not in mDA neurons), both at E15.5 and P0 (Nishikawa et al., 2003; Figures 1B,C). Based on the lack of Reelin *mRNA* in mDA neurons, Nishikawa et al. (2003) propose that Reelin might be deposited in the ventral midbrain through axonal transport in projections from the striatum to the SNc. *Dab1* is expressed in laterally positioned mDA neurons (presumptive mDA neurons of the SNc) at E13.5 and expression is maintained in a subset of mDA neurons at least up to P15 (Bodea et al., 2014; Sharaf et al., 2014; Figures 1B,C). High levels* of Dab1* expression have also been detected in the forming of substanta nigra pars reticulata (SNr; Bodea et al., 2014; Allen Institute for Brain Science, 2015). Beginning at E13.5, Reelin recepto*rs ApoER2* and *VLDLR* are both weakly expressed throughout the ventral midbrain (Bodea et al., 2014; Figures 1B,C). This widespread expression seems to be maintained at subsequent embryonic stages. At E16.5, VLDLR is expressed in and adjacent to mDA neurons, while ApoER2 expression is reported to be weaker and more specific to mDA neurons (Sharaf et al., 2014). A similar expression pattern has been documented at P15 (Sharaf et al., 2014).

### Function of Reelin Signaling in the Ventral Midbrain

Despite the broad expression of Reelin, Dab1 and the Reelin receptors in the ventral midbrain, the impact of Reelin signaling has only been studied in the context of mDA neuronal migration in the ventral midbrain and we will summarize these findings in the following section.

### Midbrain Dopaminergic (mDA) Neurons

mDA neurons in the SNc project to the dorsal striatum and form the nigrostriatal pathway; they are involved in the regulation of motor control. VTA-mDA neurons innervate the ventral striatum and prefrontal cortex, and modulate cognitive and reward behaviors. Neurons of the RRF have been reported to project to the dorsal striatum and prefrontal cortex, but their innervation targets and functions are incompletely understood (Björklund and Dunnett, 2007).

mDA neurons are generated from progenitors in the floor plate of the ventral midbrain between E10.5 and E13.5. From their progenitor zone, mDA neurons first migrate radially, towards the pial surface. Subsequently, neurons of the SNc (but not the VTA) undergo tangential migration to take up their final lateral position (Bodea et al., 2014; Figure 1D). In the absence of Reelin signaling, the SNc and RRF are disorganized, a phenotype that is first obvious at E16.5. The defect is particularly severe at intermediate anteroposterior levels in the midbrain where the lateral SNc essentially fails to form (Nishikawa et al., 2003; Kang et al., 2010; Sharaf et al., 2013; Bodea et al., 2014; Figure 1E). Consequently, there is a drastic decrease in the number of mDA neurons in the SNc at P0 compared to heterozygous controls, while there is no significant change in the overall number of mDA neurons (Nishikawa et al., 2003; Kang et al., 2010). In addition, the number of mDA neurons is significantly increased in the VTA of *reeler* mice (Kang et al., 2010). Analysis of* yotari* mice, which are homozygous for an autosomal recessive mutation in the *Dab1* gene (Sheldon et al., 1997), shows an abnormal organization of the SNc comparable to the one observed in *reeler* mutants.

Since radial and tangential fiber tracts might serve as guides for migrating mDA neurons, two studies examined the effect of the loss of Reelin signaling on these fibers. Radial glia fibers appear to be normal at E14.5 and E15.5, but are reduced at E16.5 in *reeler* mice (Nishikawa et al., 2003; Kang et al., 2010). Nishikawa et al. (2003) report unaltered tangential fiber formation (potential axonal tracts) in E15.5 *reeler* mutants, while Kang et al. (2010) show that tangential fibers are already reduced at E14.5, at least at posterior midbrain levels. These data indicate that Reelin might regulate migration of SNc-mDA neurons both in a direct manner and indirectly through regulating the normal development of guidance structures for these neurons.

The analysis of *ApoER2* and *VLDLR* single and double knockout mice demonstrates that Reelin signaling in the ventral midbrain is primarily transmitted through these canonical Reelin receptors (Sharaf et al., 2013; Bodea et al., 2014). Sharaf et al. (2013) analyzed *ApoER2* and *VLDLR* single knockout mutants, as well as *ApoER2/VLDLR* double knockout mice at P25. *ApoER2/VLDLR* double knockout show a severe reduction in the number of mDA neurons in the SNc, while *ApoER2* and *VLDLR* single knockout mice both show a mild reduction of SNc-mDA neurons, indicating that the two receptors cannot fully compensate for each other’s function. The total number of mDA neurons remains unchanged in *VLDLR* knockout mice at P25 (the numbers have not been assessed for the *ApoER2* single or *ApoER2/VLDLR* double knockout mice), in agreement with what has been reported for *reeler* and *yotari* mice (Kang et al., 2010). Consistent with the normal number of mDA neurons, cell death is not increased in mDA neurons of postnatal *ApoER2/VLDLR* double knockouts or single receptor mutants. Based on the analysis of *ApoER2/VLDLR* double knockouts at E18 and P15, Sharaf et al. (2013) report the phenotype in these mice as similar to that of the *yotari* mice (Kang et al., 2010; Sharaf et al., 2013). In contrast, the direct comparison of midbrain sections of *reeler*, *Dab1* knockout and *ApoER2/VLDLR* double knockout mice suggests that the disorganization of mDA neurons in the receptor knockout mice is less severe than in *reeler* or *Dab1* mutant mice (Bodea et al., 2014). Thus, additional, non-canonical Reelin receptors might be involved in transducing the signal.

Calbindin and Girk2 (G protein-gated inwardly rectifying potassium channel 2 also known as Kcnj6) label two distinct mDA neuronal subpopulations; Calbindin positive cells are primarily located in the VTA, Girk2 positive cells predominantly in the SNc. Girk2 positive mDA neurons are still located adjacent to the Calbindin-positive VTA-mDA neurons at P25 in *reeler*, *Dab1* and *ApoER2/VLDLR* knockout mice (Bodea et al., 2014; Figure 1E). Similarly, Calbindin negative mDA neurons are mostly found lateral to Calbindin positive mDA neurons in the VTA of *VLDLR* single knockout mice (Sharaf et al., 2013). Hence, Reelin signaling does not affect the segregation of SNc and VTA neurons. Together, these data indicate that in the absence of Reelin signaling SNc-mDA neurons fail to migrate out to their normal lateral positions and remain clustered medially, adjacent to the VTA. Despite the disorganization of SNc- and RRF-mDA neurons, nigrostriatal projections show no obvious alterations in *reeler* mutant, *Dab1* knockout or *ApoER2/VLDLR* double knock-out mice (Nishikawa et al., 2003; Sharaf et al., 2013).

Given the abnormal distribution of SNc-mDA neurons in mice in which the Reelin signaling pathway is inactivated, Bodea et al. (2014) studied how Reelin signaling alters the migratory behavior of SNc-mDA neurons. Migrating mDA neurons were monitored with time-lapse imaging in an organotypic slice culture system. Blocking Reelin signaling with a function-blocking antibody for Reelin in these slices results in decreased speed of tangentially migrating neurons, while speed of radially migrating neurons is not affected. In addition, inhibiting Reelin signaling in slices causes a significant deviation of migrating neurons from tangential trajectories as compared to untreated slices. Accordingly, analysis of laterally positioned mDA neurons in E13.5 *reeler* mice show that these mDA neurons fail to orient tangentially and are instead oriented perpendicular to their direction of migration (Figures 1D–D″).

Despite these advances, it remains to be elucidated whether Reelin is required directly by migrating mDA neurons or influences mDA neuronal migration by altering guidance scaffolds. While it is clear that Reelin is involved in the later part of tangential migration of SNc-mDA neurons, the mechanism by which the Reelin signal regulates this process, and the downstream factors involved are yet to be unraveled.

## Ventral Hindbrain

The ventral hindbrain consists of a large array of anatomically and functionally distinct neuronal clusters. The embryonic hindbrain is divided into eight rhombomeres (r1–r8) along its rostrocaudal axis. Hindbrain neurons are generated in the ventricular zone and a specialized germinal zone at the dorsal tip of the hindbrain, the rhombic lip. These neurons undergo complex migration events along the dorsoventral and rostrocaudal axis that occur over several days during embryonic development (Ray and Dymecki, 2009; Wanner et al., 2013). Thus, the final location of a particular neuronal cluster along the rostrocaudal or dorsoventral axis of the hindbrain is not necessarily indicative of its origin. As mentioned in the introduction, we focus our discussion on the hindbrain cranial motor neurons since they originate from the ventral progenitor domain in the hindbrain.

### Expression Pattern of Reelin and Its Downstream Pathway Components in the Ventral Hindbrain

**Figure 2:**
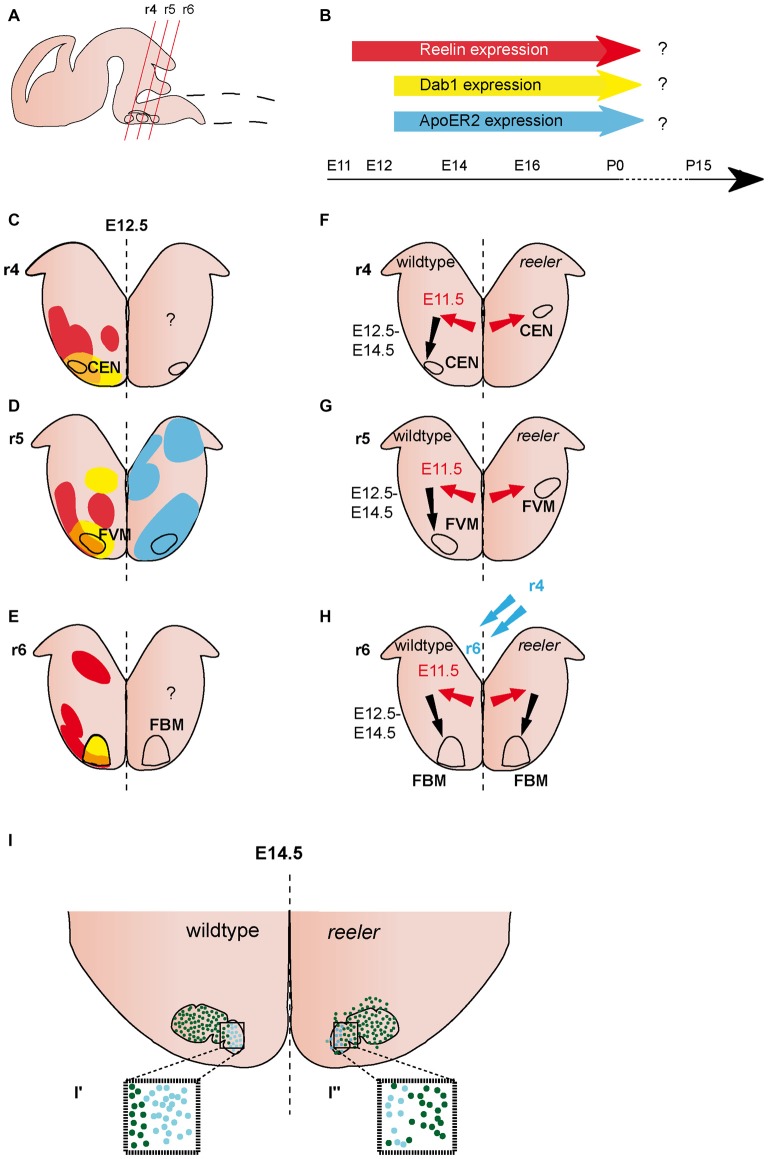
**Migration of ventrally derived hindbrain neurons. (A)** Sagittal view of embryonic brain. Red lines indicate level of coronal section in **(C–H)**. **(B)** Expression of Reelin, Dab1 and ApoER2 over time. **(C–E)** Expression patterns of Reelin (red), Dab1 (yellow) and ApoER2 (blue) at the indicated time point. Reelin and Dab1 expression are only presented in the left half of the brain, ApoER2 expression is only presented in the right half of the brain. Question mark indicates a lack of expression data. The VLDLR expression pattern is not well characterized in the hindbrain, thus it is not included in the schematics in **(B–E)**. **(F–H)** Schematics of migratory paths of cochlear efferent nucleus (CEN) neurons, facial visceral motor (FVM) neurons and facial branchial motor (FBM) neurons in the wildtype (left half of the brain) and *reeler* mutants (right half of the brain). Red arrows indicate dorsolateral tangential migration, black arrows indicate ventral radial migration, and blue arrows indicate rostrocaudal migration. **(F,G)** Ventral migration of CEN and FVM neurons is truncated in *reeler* mutants. **(H,I)** The majority of FBM neurons reach their final superficial position in r6 of the hindbrain, but the nuclei shows subtle disorganizations. **(I^′^,I^″^)** The medial lobe (Lhx4+) is reduced (turquoise dots), the lateral lobe (Er81+) is disorganized (dark green dots) in *reeler* mutants compared to the wildtype.

Reelin expression is observed in the lateroventral hindbrain at E12.5 (Ikeda and Terashima, 1997; Rossel et al., 2005; Allen Institute for Brain Science, 2015). Expression appears in patches, suggesting that Reelin is expressed in specific clusters of cells. These patches are mostly juxtaposed to *Dab1* positive areas, but there also appears to be some overlap of the two expression patterns (analyzed in rhombomere r4–r6; Rossel et al., 2005; Figures 2A–E). At E14.5, *Reelin* is widely expressed in the ventrolateral hindbrain, but appears to be excluded from certain cell populations, including the facial motor neurons (Ikeda and Terashima, 1997; Carroll et al., 2001; Allen Institute for Brain Science, 2015). This broad expression pattern appears to be maintained at later embryonic stages (Allen Institute for Brain Science, 2015). *Dab1* expression can be detected at E11.5 (Allen Institute for Brain Science, 2015). Starting at E12.5, it is expressed in several cell clusters in the ventral hindbrain, including the facial branchial motor (FBM) nucleus and the facial visceral motor (FVM) nucleus (Carroll et al., 2001; Rossel et al., 2005; Allen Institute for Brain Science, 2015; Figures 2A–E). *ApoER2* is also widely distributed in the developing hindbrain, but it remains unclear whether it is expressed in the neuronal populations (see below) that are altered in the absence of Reelin signaling. The expression onset is after E11.5 (Rossel et al., 2005; Allen Institute for Brain Science, 2015; Figures 2A–E). The expression of *VLDLR* has essentially not been assessed in the hindbrain, except for the r5 level, where it is not or only very weakly expressed at E12.5 (Rossel et al., 2005).

### Function of Reelin Signaling in the Ventral Hindbrain

#### Trigeminal Motor Nucleus

The trigeminal motor nucleus (motor nucleus of V) in r2 and r3 extends projections to the muscles of the first branchial arch that control the jaw jerk reflex. Trigeminal branchiomotor neurons are generated in the ventricular zone of ventral r2 and r3 between E9.5 and E10.5. Upon exiting the cell cycle they migrate dorsolaterally to their final position in the trigeminal motor nucleus (Pattyn et al., 2003; Ohsawa et al., 2005). Terashima et al. (1994) used horseradish peroxidase (HRP) injections into jaw-opening or jaw-closing muscles of wildtype and *reeler* mice to retrogradely label the subsets of trigeminal motor neurons that control these different muscles. They demonstrated that the motor neurons that control jaw-opening muscles are more scattered in *reeler* mice compared to wild-type. Whether this defect is caused by aberrant neuronal migration in the absence of Reelin signaling has not been investigated.

#### Facial Motor Neurons

The facial nucleus (nucleus VII) is comprised of branchial and visceral motor neurons (FBM and FVM neurons) that innervate muscles controlling facial expressions and parasympathetic ganglia, respectively. The FBM neurons are generated in the ventral ventricular zone of r4 and r5, the FVM neurons in the ventral ventricular zone of r5. After leaving the ventricular zone after E10.5, FVM neurons migrate first dorsolaterally within r5 and then towards the pial surface to form the the superior salivatory nucleus. FBM neurons migrate caudally along the ventricular surface to r6 (or r5 in avian species) and then dorsolaterally and radially to form the facial nucleus. Both nuclei are in a superficial position, close to the pial surface (Goffinet, 1984a; Garel et al., 2000; Jacob and Guthrie, 2000; Guthrie, 2007; Wanner et al., 2013).

The organization of the FBM neurons in the facial nucleus is altered in *reeler* mutants (Goffinet, 1984a; Terashima et al., 1993; Rossel et al., 2005). Based on morphological analysis and labeling with the motor neuron marker *Isl1 (Islet-1)*, the initial steps of migration appear not to be affected in *reeler* mutants, since no difference in the morphology, clustering or positioning of the presumptive FBM neurons was observed between wildtype and *reeler* mutants at E11.5 or E12.5 (Goffinet, 1984a; Rossel et al., 2005). By E14.5, when the migration of FBM neurons is largely complete, the large majority of FBM neurons reach their final superficial position in *reeler* mutants, but FBM neurons are more scattered than in wildtype brains (Goffinet, 1984a). At this stage, the facial nucleus is organized into a medial and a lateral lobe. Analysis of markers that label either the lateral lobe, Etv1 (Ets variant gene 1 also known as Er81) or medial lobe, *Lhx4* (Lim homeobox protein 4) demonstrated that the medial lobe is reduced in size while neurons in the lateral lobe seem to be more scattered in *reeler* mutants as compared to wildtype (Rossel et al., 2005; Figures 2H,I). By E17.5, the wildtype facial nucleus is divided into several different anatomical subsets in the wildtype brain, this segregation is less obvious in the *reeler* mutants (Goffinet, 1984a). Whether Reelin affects the late migration steps of FBM neurons or other aspects of their maturation has not been assessed (Figures 2H,I). Despite the apparent disorganization of the facial motor nucleus in *reeler* mutants, the topographic representation of the muscle targets appears to be preserved in the nucleus as shown by HRP-based retrograde tracing from the muscles innervated by the facial nerve (Terashima et al., 1993).

In contrast to the FBM neurons, the FVM neurons do not reach their final superficial position in the *reeler* mutants. Analysis of the hindbrain motor neuron markers *Isl1*, *Ret* (RET proto-oncogene) and *Phox2B* (paired like homeobox 2b) shows ectopic neuronal clusters in the lateral hindbrain that are either positioned close to the ventricular zone or in an intermediate position (between ventricular zone and pial surface; Rossel et al., 2005; Figure 2G). DiI labeling of cell bodies and projections of FVM neurons at E11.5 and E12.5 demonstrates that the FVM neurons are born at the correct time point and reach a lateral position. However, in wildtype embryos, the FVM neurons reach the pial surface by E12.5, while they remain deeper in the hindbrain tissue in the *reeler* mutants (Rossel et al., 2005; Figure 2G). These data suggest that FVM neurons undergo normal lateral migration in the absence of Reelin signaling, but are not able to relocate towards the pial surface. Radial glia fibers appear to be normal in the hindbrain of *reeler* mice (Rossel et al., 2005), suggesting that Reelin signaling affects the migration of these neurons directly. Whole-mount analysis of *Isl1* expressing motor neurons in the hindbrain of *scrambler* mutants, which are null mutants for *Dab1*, shows that the *scrambler* phenotype is comparable to the one in *reeler* mutants. In contrast, the *ApoER2/VLDLR* double knockout mice have no apparent phenotype (Rossel et al., 2005). Further analysis will be necessary to assess the cause for this discrepancy. In particular it should be investigated whether the canonical Reelin receptors are expressed in migrating FBM and FVM neurons.

#### Cochlear Efferent Nucleus

The neurons of the cochlear efferent nucleus (CEN) are generated from the motor neuron progenitor domain in r4. After leaving the progenitor area between E10.5 and E12.5, the differentiated neurons are initially intermingled with the facial brachial motor neurons. Subsequently, the two populations segregate and the neurons of the CEN take a dorsolateral migratory route within r4 where they settle close to the pial surface of the lateral hindbrain by E14.5 (Bruce et al., 1997; Rossel et al., 2005; Figure 2F). Analysis of the position of CEN neurons in E12.5 wildtype and *reeler* mutant brains with retrograde labeling, and the markers *GATA3* (Gata binding protein 3) and *Tbx20* (T-box 20) shows that similar to the FVM neurons, CEN neurons do not reach their final superficial position. Instead, ectopically clustered cells are observed close to the ventricular zone in a lateral position where they should normally initiate their migration towards the pial surface. As described above for the FVM neurons, CEN neurons appear to be unable to reach their final position in *scrambler* mutants, but appear not to be affected in *ApoER2/VLDLR* double mutants (Rossel et al., 2005).

#### Ambiguus Nucleus

The branchimotor neurons of nucleus IX are clustered in the nucleus ambiguus in r6 of the hindbrain and innervate the esophagus, larynx, pharynx and palate. These neurons are important regulators of swallowing and speech. Little is known about their development in the rodent model, but analysis of human embryonic and fetal brains suggests that their migration path is similar to the one observed for trigeminal or FVM neurons in rodents (Brown, 1990; Figure 2G). Retrograde tracing with a lacZ-expressing adenoviral vector has been used to label the branchimotor neurons that project to the esophagus. While these neurons are tightly clustered in a lateral position close to the pial surface in the wildtype hindbrain, they are located deeper within the lateral hindbrain tissue in the *reeler* mutant mice, suggesting that their final migration step towards the pial surface could be altered in the absence of Reelin signaling (Fujimoto et al., 1998). The disorganization of the ambiguous nucleus (and of the trigeminal and facial nucleus) was also described in *shaking rat Kawasaki*, a rat strain that harbors an autosomal recessive mutation in the *Reelin* gene (Setsu et al., 2001; Kikkawa et al., 2003).

In summary, the migration of branchial motor neurons involves an initial lateral migration step followed by a change in direction of migration and radial migration towards the pial surface. The phenotypes observed in the absence of Reelin signaling suggest that Reelin is involved in the latter step of migration and/or in the change in direction of migration. Reelin appears to influence the migration of these neurons directly, since the radial glia scaffold has been described as being normal in the hindbrain of *reeler* mutants.

## Spinal Cord

**Figure 3:**
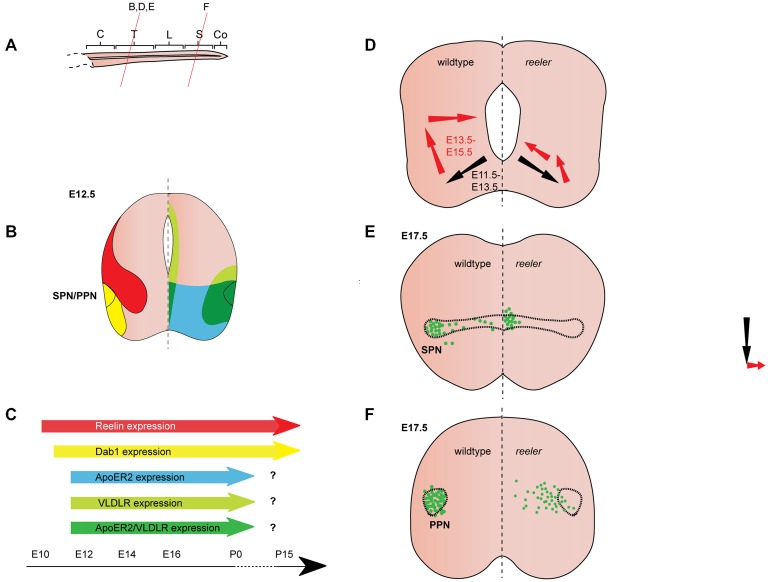
**Reelin signaling in the migration of autonomic preganglionic neurons. (A)** Sagittal view of embryonic spinal cord (C, cervical; T, thoracic; L, lumbar; S, sacral; Co, coccygeal). Red lines indicate level of coronal section in **(D–F)**. **(B)** Expression patterns of Reelin (red), Dab1 (yellow), ApoER2 (green) and VLDLR (green) at the indicated time point. Reelin and Dab1 expression are only presented in the left half of the brain; ApoER2 and VLDLR expression are only presented in the right half of the brain. **(C)** Expression of Reelin, Dab1, ApoER2 and VLDLR over time. **(D)** Schematic of migratory paths of preganglionic autonomic neurons in wildtype mice (left half of the brain) and *reeler* mutants (right half of the brain). Black arrows indicate radial migration; red arrows indicate tangential migration. Radial migration is unaffected in *reeler* mutants, but in the second step of migration the majority of preganglionic autonomic neurons migrate to an ectopic position in the medial regions of the spinal cord. **(E)** SPN distribution at E17.5 in wildtype (left half of the brain) and *reeler* (right half of the brain) mice. Sympathetic preganglionic neurons (SPNs) are ectopically located close to the central canal in *reeler* mice. **(F)** Parasympathetic preganglionic neuron (PPN) distribution at E17.5 in wildtype mice (left half of the brain) and *reeler* mutants (right half of the brain). PPN are disorganized and distributed along the mediolateral axis of the intermediate spinal cord in *reeler* mutants.

The spinal cord is divided from rostral to caudal into cervical, thoracic, lumbar, sacral and coccygeal levels (Figure 3A). Ventral spinal cord neurons are arranged into columns that are established according to their final projection targets. For example, somatic motor neurons (SMNs) that project to the limbs are organized into the lateral motor column (LMC) at brachial (lower cervical) and lumbar levels of the spinal cord. Preganglionic neurons are clustered into the preganglionic column at thoracic (intermediolateral nucleus) or sacral levels (intermediolateral sacral nucleus). SMNs, V0–V3 interneurons, and preganglionic neurons originate from progenitors in the ventricular zone in the ventral spinal cord. Subsequently, they migrate to distinct dorsoventral positions: SMNs and V3 interneurons are located ventrally, preganglionic neurons are positioned at intermediate dorsoventral levels of the spinal cord and V0–V2 neurons are distributed in positions between SMNs and preganglionic neurons (Lee and Pfaff, 2001; Gaella, 2004; Dalla Torre di Sanguinetto et al., 2008).

### Expression Pattern of Reelin and Its Downstream Pathway Components in the Spinal Cord

**Figure 4:**
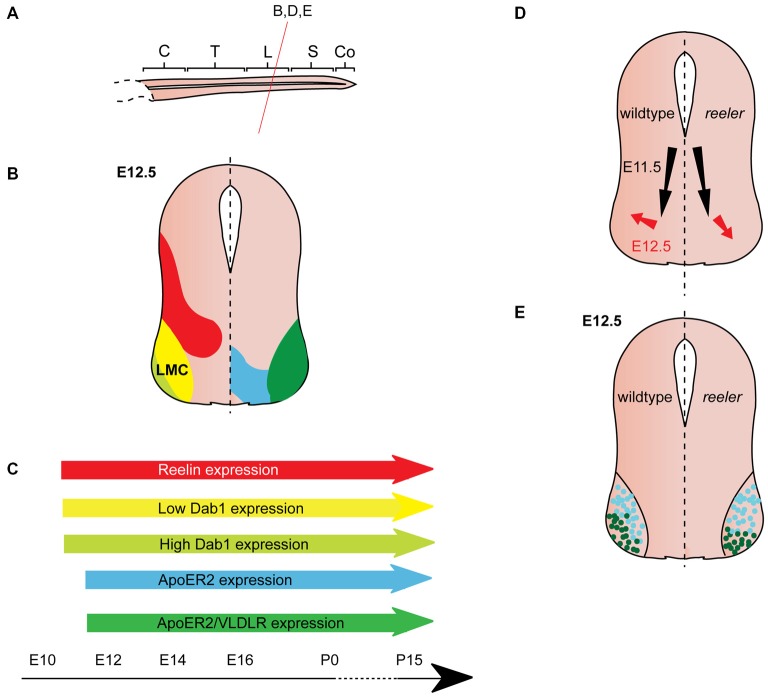
**Reelin signaling in motor neuron migration. (A)** Sagittal view of embryonic spinal cord (C, cervical; T, thoracic; L, lumbar; S, sacral; Co, coccygeal). Red line indicates level of coronal section in **(D,E)**. **(B)** Expression patterns of Reelin (red), Dab1 (low levels: yellow, high levels: light green), ApoER2 (blue) and ApoER2/VLDLR (green) at the indicated time point. Low-level Dab 1 expression is restricted to Foxp1+, Isl1+ neurons in the medial part of the lateral motor column (LMC), high-level Dab1 expression is found in Foxp1+, Lhx1+ neurons in the lateral LMC. Reelin and Dab1 expression are only presented in the left half of the brain; ApoER2 and VLDLR expression are only presented in the right half of the brain. **(C)** Expression of Reelin, Dab1, ApoER2 and VLDLR over time. **(D)** Schematic of migratory paths of somatic motor neurons (SMNs) in wildtype mice (left half of the brain) and *reeler* mutants (right half of the brain). Black arrows indicate ventral radial migration of SMNs from the neuroepithelium to the LMC; red arrows indicate a short tangential migratory step, which enables a subset of SMNs to migrate dorsolaterally to form the lateral LMC. Radial migration is unaffected in *reeler* mice, but the dorsolateral migration is altered. **(E)**. As compared to their wildtype counterparts, lateral LMC neurons (Foxp1+, Lhx1+; dark green dots) settle in more ventromedial positions in the *reeler* spinal cord. Turquoise dots represent medial LMC neurons (Foxp1+, Isl1+).

*Reelin* expression begins in the cervical region of the murine spinal cord at E9.5, when it appears to be restricted to subsets of differentiated neurons in the ventral and intermediate spinal cord (Ikeda and Terashima, 1997). By E11.5, Reelin mRNA and protein are expressed dorsally and medially to the motor column throughout the spinal cord (Ikeda and Terashima, 1997; Schiffmann et al., 1997; Yip et al., 2004a; Palmesino et al., 2010; Lee and Song, 2013). Between E11.5 and E12.5, Reelin protein is expressed in ventral and intermediate regions of the spinal cord but is excluded from SMNs and preganglionic neurons (Yip et al., 2000, 2004a; Phelps et al., 2002; Palmesino et al., 2010; Lee and Song, 2013; Figures 3B,C, 4B,C). A similar pattern is observed in chick at E4.5 and E6.5 (Palmesino et al., 2010). Since Reelin protein is deposited around the cell bodies of V1 and V2 interneurons at this stage, it has been suggested that they are the source of Reelin (Yip et al., 2004b). Reelin expression is maintained in the ventral and intermediate spinal cord at subsequent stages of embryonic development. Starting between E13.5 and E14.5, Reelin can be also detected in a band of cells in the dorsal superficial horn (Phelps et al., 2002; Kubasak et al., 2004; Villeda et al., 2006) and in the ventricular zone at intermediate dorsoventral levels (Ikeda and Terashima, 1997; Phelps et al., 2002; Hochstim et al., 2008). The onset of expression in the ventricular zone appears to coincide with the onset of gliogenesis (Hochstim et al., 2008). In the prenatal and adult spinal cord, *Reelin* mRNA is expressed in the intermediate gray, the superficial dorsal horn and in a subset of white matter astrocytes in the lateral funiculus (Ikeda and Terashima, 1997; Phelps et al., 2002; Kubasak et al., 2004; Hochstim et al., 2008). The Reelin expression pattern is largely conserved between rat and mouse. In chick spinal cord, Reelin is already expressed by E4 and the Reelin expression pattern is comparable to the patterns observed in rodents (Bernier et al., 2000; Kubasak et al., 2004; Palmesino et al., 2010).

From E10.5 onwards, Dab1 mRNA and protein is expressed in the lateral intermediate and ventral region in the spinal cord, where it colocalizes with different neuronal populations, depending on the rostrocaudal level (Phelps et al., 2002; Yip et al., 2004a; Palmesino et al., 2010; Lee and Song, 2013). At E12.5, Dab1 protein expression in the somatic motor column is weak at upper cervical levels, but strong at brachial, thoracic and lumbar levels (Palmesino et al., 2010; Lee and Song, 2013). Within the LMC, Dab1 is highly expressed in the lateral LMC subpopulation characterized by the expression of Foxp1 (forkhead box protein 1) and Lhx1 (LIM homeobox 1), while Dab1 is weakly expressed in the dorsomedial LMC subpopulation expressing Foxp1 and Isl1 (Palmesino et al., 2010; Figure 4B). In the chick, Dab1 also colocalizes with Lhx1-positive SMNs at E4.5 and E6.5 (Palmesino et al., 2010). Dab1 protein colocalizes with migrating sympathetic preganglionic neurons (SPNs) in the intermediolateral nucleus at thoracic levels and with migrating parasympathetic preganglionic neurons (PPNs) at sacral levels at E12.5 (Phelps et al., 2002; Yip et al., 2004a; Figure 3B). SPNs, PPNs and lateral LMC neurons continue to express *Dab1* throughout development and well into adulthood (Abadesco et al., 2014). *ApoER2* is expressed throughout the ventral region of the spinal cord at thoracic levels at E12.5, while *VLDLR* expression is restricted to the lateral intermediate region (Yip et al., 2004a; Figure 3B). At the lumbar level, *ApoER2* is expressed in the ventricular zone and in LMC neurons in mouse at E11.5 (Palmesino et al., 2010; Figure 4B). In the chick spinal cord, *ApoER2* is only expressed in the ventricular zone at E4.5 and E6. In the mouse, VLDLR protein expression at E11.5 is higher in the lateral LMC neurons than in their medial counterparts. By E12.5, VLDLR is uniformly expressed in the LMC. VLDLR expression is also observed in chick embryos at E4 and E6.5 (Palmesino et al., 2010).

### Function of Reelin signaling in the Ventral Spinal Cord

Throughout the spinal cord, Reelin and Dab1 expression appear to be mutually exclusive. Neurons of the LMC, SPNs, and PPNs are Dab1-positive and express either one or both Reelin receptors. This suggests that these Dab1-expressing neurons require Reelin signaling for their correct positioning, while populations expressing Reelin but not Dab1, such as the V1 and V2 interneurons, do not require Reelin signaling for their migration (Phelps et al., 2002; Yip et al., 2004b). In the following sections, we discuss the role of Reelin signaling in the migration of SPNs, PPNs, and neurons in the LMC.

### Sympathetic Preganglionic Neurons (SPNs)

SPNs are acetylcholine-releasing neurons located in the intermediolateral column of the thoracic spinal cord. They project to pre- and paravertebral ganglia where they innervate postganglionic autonomic motor neurons, which in turn innervate visceral organs and neuroendocrine systems (Yip et al., 2000; Gaella, 2004). Beginning at E11.5 in the mouse, SPNs migrate radially from the ventral neuroepithelium towards the ventrolateral regions of the forming mantle layer. Together with SMNs they establish a rudimentary motor column. In the second step of their migration, SPNs separate from the SMNs by migrating dorsally in the lateral spinal cord. Once they reach the intermediate dorsoventral levels of the spinal cord they form the intermediolateral column. A small subset of SPNs undergoes further migration towards medial regions and settles close to the central canal. By E15.5, SPN migration is essentially complete (Phelps et al., 1991, 2002; Yip et al., 2000, 2003; Figure 3D). In *reeler* mice, a large number of SPNs are localized close to the central canal, while very few neurons are observed in the intermediolateral column (Yip et al., 2000; Phelps et al., 2002; Figure 3E). In addition, some SPNs are located outside of the spinal cord in *reeler* mice (Yip et al., 2003). *Dab1* knockout and* ApoER2*/*VLDLR* double knockout mice have a *reeler* like phenotype. *VLDLR* or *ApoER2* single knockout mice have no apparent phenotype, suggesting that the receptors can compensate for each other’s function (Yip et al., 2004b). Monitoring the position of migrating SPNs at half-day intervals between E11.5 and E15.5 in wildtype and *reeler* mice, Yip et al. (2003) showed that the initial radial migration takes place normally in the mutant mice. At E12.5, when SPNs initiate their dorsolateral migration, SPNs in the wildtype orient themselves parallel to the dorsoventral axis, while SPNs in *reeler* mutants show a medial or a dorsomedial orientation. Consistent with this abnormal orientation, SPNs in E13 *reeler* mutant mice migrate in a dorsomedial direction (parallel to radial glia fibers) towards the central canal, instead of migrating dorsolaterally to form the intermediolateral column (Yip et al., 2003; Figure 3D).

In the chick, the final position and migration of SPNs differ from the one described in mouse: after moving to the ventrolateral spinal cord, SPNs migrate close to the ventral midline to reach a more dorsal position. Once they arrive at the intermediate spinal cord they settle in the column of Terni next to the central canal. During this dorsally directed migration, the neurons appear to avoid Reelin rich areas in the lateral spinal cord. Misexpression of Reelin in the migratory path of dorsally-migrating SPNs leads to the stalling of these neurons ventral to the Reelin expressing cells (Yip et al., 2011). These data suggest that Reelin acts as a repellent for dorsally migrating SPN neurons in the chick.

There is also evidence for Reelin as a repellent in murine SPNs. To test whether ectopic expression of Reelin can rescue the defective SPN migration in *reeler* mutants, Yip et al. (2009) analyzed *reeler* mutants, in which Reelin is misexpressed under the control of the *Nestin* promoter (referred to as *reeler-NeReelin* mice; Magdaleno et al., 2002; Yip et al., 2009). In these mice, Reelin is strongly expressed in the neuroepithelium at the midline of the spinal cord starting at E9.5, but it is still absent from the areas where Reelin is normally expressed in the wildtype. The ectopic expression of Reelin does not affect the initial migration of SPNs to the ventrolateral spinal cord in the *reeler-NeReelin* mice, but SPNs still undergo abnormal dorsally-directed migration. This is evident in a lateral-to-medial shift in the location of SPNs. The SPNs in the *reeler-NeReelin* mice are distributed between their normal lateral position and the central canal up to E16.5. After E18.5 neurogenesis ceases in the spinal cord and with the disappearing neuroepithelium the midline expression of Reelin vanishes. SPNs in E18.5 and postnatal *reeler-NeReelin* mice are localized in two clusters, one in the intermediolateral column and one close to the central canal. Thus, the phenotype in the *reeler-NeReelin* mice is milder than in the *reeler* mice (Yip et al., 2009; Figure 3E), suggesting that the ectopic expression of Reelin in the neuroepithelium results in a partial rescue of the *reeler* phenotype. The fact that a subset of SPNs only accumulates close to the central canal after Reelin expression ceases in the midline, has been interpreted as an indication for a repellent function of Reelin in the migration of SPNs. A repellent function of Reelin would be consistent with the observation that ventrolaterally located SPNs are separated from V1 and V2 interneuron clusters (a likely source of Reelin protein) in E12.5 wildtype mice, while SPNs and interneurons are intermingled in *reeler* mice (Yip et al., 2004b). In addition, several other hypotheses have been posited regarding the function of Reelin in migration of murine SPNs. Reelin signaling might sensitize migrating SPNs to attractive cues in the intermediolateral column (Yip et al., 2003). Alternatively, Reelin signaling could affect SPN migration by facilitating the correct orientation of the neuronal cell bodies, since secreted Reelin can be detected laterally and directly adjacent to migrating SPNs (Kubasak et al., 2004). Such a role for Reelin signaling would be consistent with one of the known functions of Reelin in cortical migration (Jossin and Cooper, 2011). Radial glia fibers are not grossly altered in the spinal cord of *reeler* mice (Yip et al., 2003; Lee and Song, 2013) but Reelin might act by modulating the attachment of SPNs to radial glia fibers (Kubasak et al., 2004).

As in the cerebral cortex, Reelin signaling in the spinal cord requires the phosphorylation of Dab1 via Src/Fyn tyrosine kinases (Howell et al., 1997; Hiesberger et al., 1999; Arnaud et al., 2003; Yip et al., 2007). A *reeler*-like phenotype has been reported in the SPNs of *Src*/*Fyn* double knockout mice and in mice that are homozygous for a null allele of *Dab1* (*Dab1^lacZ/lacZ^*) or that express a Dab1 protein in which the tyrosine phosphorylation sites are abolished (*Dab1^5F/5F^* mice; Howell et al., 2000; Yip et al., 2007; Abadesco et al., 2014). Src and Fyn might be able to compensate for each other’s function, as the single mutants do not fully mimic the *reeler* phenotype (Yip et al., 2007). Phosphorylated Dab1 can recruit the adaptor molecules Crk, CrkL and CrkII and this interaction has been shown to regulate cell adhesion properties of migrating cortical neurons via the Crk/CrkL-C3G-Rap1 pathway (Ballif et al., 2004; Franco et al., 2011). To investigate whether this pathway acts downstream of Dab1 in the spinal cord, Yip et al. (2007) analyzed the development of SPNs in *Crkl* and* C3G* mutants. In *CrkL* knockout mice, some SPNs were still located in the intermediolateral column, but the majority of SPNs were clustered close to the central canal or were interspersed between the central canal and the intermediolateral column. The phenotype in *CrkL* knockout mutant mice appears to be milder than the one observed in *reeler* mutants (Figure 3E), thus Crk might be able to compensate for the loss of CrkL. In mice homozygous for a hypomorphic allele of C3G (*C3G^gt/gt^*) SPNs are scattered between the intermediolateral column and the central canal. This phenotype is less severe than in the *reeler* mutants, suggesting that there are other downstream pathways or that a small amount of C3G protein in the hypomorphs is sufficient for residual downstream signaling (Yip et al., 2012).

The actin binding protein Cofilin1, which is an actin depolymerizing protein, may be another factor acting downstream of Reelin. Activation of Reelin signaling results in the LIM kinase-dependent phosphorylation of Cofilin1 (p-Cofilin1) and the attenuation of the actin-depolymerizing activity of Cofilin1. Thus, the phosphorylation of Cofilin1 contributes to the stabilization of the actin cytoskeleton (Chai et al., 2009). p-Cofilin1 is strongly expressed in the intermediolateral column of wild-type mice at E13.5 (Krüger et al., 2010). In contrast, almost no p-Cofilin1 expression is detected in the SPNs of *reeler* or* Dab1* knockout mice at this time point. Low-level p-Cofilin1 labeling is observed in *ApoER2* knockout mice while *VLDLR* knockout mice show weak but more distributed p-Cofilin1 expression compared to the wildtype. Moreover, the level of p-Cofilin1 increases when spinal cord tissue of *reeler* mutant mice is treated with recombinant Reelin protein (Krüger et al., 2010). This evidence suggests that Reelin plays a role in the stabilization of the cytoskeleton in migrating SPNs via p-Cofilin1, possibly causing their migratory arrest.

### Parasympathetic Preganglionic Neurons

Parasympathetic preganglionic neurons (PPNs) at the sacral levels of the spinal cord are located in the intermediolateral sacral nucleus. These neurons are cholinergic and project over long distances to synapse on their postganglionic targets, which in turn innervate non-voluntary muscles and neuroendocrine glands. PPNs migrate in a similar fashion to SPNs: first radially towards a ventrolateral position and then tangentially (dorsally) to form the intermediolateral sacral nucleus. PPNs are laterally clustered, but unlike the SPNs, are completely absent from medial regions (Phelps et al., 2002). In *reeler or Dab1^lacZ/lacZ^* mice, PPNs are disorganized and dispersed along the mediolateral axis of the intermediate region of the sacral spinal cord (Figure 3F). Expression patterns of Reelin and Dab1 are similar at thoracic and sacral levels (Figure 3B) and most of the mechanisms proposed for SPN migration have been extended to PPNs (Phelps et al., 2002; Kubasak et al., 2004; Abadesco et al., 2014).

### Somatic Motor Neurons

SMNs are located in the motor column of the ventrolateral spinal cord and are found at all rostrocaudal levels of the spinal cord. Their projections exit through the ventral root of the spinal cord, innervate skeletal muscle, and regulate voluntary movement. SMNs are born in the ventral neuroepithelium from where they migrate along radial glia fibers towards the ventrolateral mantle layer along with preganglionic neurons (Phelps et al., 1991). After their radial migration has ended, they maintain more or less the same position, and undergo only a small tangential migration step to arrange themselves in their final position (Palmesino et al., 2010).

SMN migration was generally believed to be normal in the absence of Reelin signaling (Phelps et al., 2002) until a study reported that Reelin is required for correct positioning of LMC neurons at lumbar-sacral levels, where SPNs and PPNs are absent (Palmesino et al., 2010; Figure 4A). Delineation of a medial subpopulation (Foxp1-positive, Isl1-positive, low-level Dab1 expression) from a lateral subpopulation (Foxp1-positive, Lhx1-positive, high-level Dab1 expression) reveals subtle defects in the positioning of these LMC neuronal subsets in *Dab1* knockout mice and *reeler* mice (Palmesino et al., 2010; Figures 4B,C). The initial radial migration of LMC neurons is normal in the absence of Reelin signaling. However, the tangential migration of these neurons is altered: the high-level Dab1-expressing, lateral LMC subpopulation is shifted to a more medioventral position in the mutants (Figures 4D,E). These findings are further corroborated by the analysis of *lacZ*-expressing SMNs (corresponding to the Dab1-expressing lateral LMC neurons) at lumbar levels of the adult spinal cord of *Dab1^LacZ/LacZ^* mice showing that the position of lateral LMC neurons is shifted medially and ventrally (Abadesco et al., 2014). Despite the disorganization of the LMC in the absence of *Dab1*, projections of medial and lateral LMC follow their normal trajectories in the ventral and dorsal limb nerve, respectively (Palmesino et al., 2010). In contrast, dendrites of lateral LMC neurons that project towards the lateral funiculus appear to be reduced and disorganized (Abadesco et al., 2014).

When Dab1 is misexpressed in LMC neurons in chick lumbar spinal cord at E6, the lateral subpopulation, which already expresses a high level of Dab1, is unaltered in position, but the medial subpopulation, which normally expresses low levels of Dab1, is shifted to a more lateral position (Palmesino et al., 2010). These data suggest that Reelin signaling promotes LMC neuronal migration directly, or enables LMC neurons to sense cues required for their lateral migration. As the medial subpopulation of LMC neurons expresses low levels of Dab1, the authors hypothesize that Dab1 is quickly degraded after phosphorylation downstream of Reelin signaling and hence results in their migratory arrest. Lateral LMC neurons migrate more laterally due to their high level of Dab1 expression.

How are the different expression levels of Dab1 in the two LMC subpopulations regulated? Analysis of transgenic and knockout mice showed that Foxp1 positively controls the expression of Dab1 in motor neurons: misexpression of Foxp1 in all motor neurons leads to abnormally high expression of Dab1 in motor neurons at upper cervical, where Foxp2 and Dab1 are normally absent or expressed at very low levels (Palmesino et al., 2010). Concordantly, in *FoxP1* knockout mice, Dab1 expression is reduced in motor neurons at lower cervical levels, where Foxp2 and Dab1 are expressed at high levels in the wildtype. Despite these results, it is unlikely that Foxp1 determines the different levels of Dab1 expression in medial and lateral LMC neurons, since it is expressed in both populations. Isl1 and Lhx1 determine the fate of medial and lateral LMC neurons, respectively, and are thus more likely regulators of Dab1 expression levels in the two LMC subpopulations. Indeed, Isl1 misexpression downregulates Dab1 expression, while Lhx1 misexpression upregulates Dab1 expression. Consistent with these results, conditional inactivation of *Lhx1* in the LMC neurons leads to decreased Dab1 levels in lateral LMC and a medial clustering of these neurons (Palmesino et al., 2010).

Finally, at brachial and thoracic levels of the spinal cord in *reeler* mutants, ectopic Foxp1-positive, Isl1-positive LMC neurons and medial motor column neurons are found outside the spinal cord (Lee and Song, 2013). Since Reelin is localized to radial glia end feet, the authors propose that Reelin might inhibit motor neurons from migrating out of the spinal cord, while allowing axonal efferents to exit the spinal cord and reach target areas. Hence Reelin may be involved in regulating two independent aspects of motor neuron migration: positioning lateral LMC neurons and hindering motor neurons from migrating out of the spinal cord.

## Conclusion

In conclusion, the common role of Reelin in the migration of neurons in the ventral brain stem and spinal cord seems to be restricted to the late or final step of neuronal migration. Interestingly, Reelin regulates both tangential (midbrain and spinal cord) and radial migration (hindbrain). How Reelin influences the behavior of these migrating neurons is still not completely understood and several possible roles have been suggested: Reelin might act as an attractant, repellent, or permissive factor. Since Reelin appears to influence only the second or third step of migration and leads to changes in tangential as well as radial migration routes, it is tempting to speculate that the main function of Reelin might be in changing the orientation of migrating neurons at the time when they have to alter the direction of their migratory route. This change in orientation could possibly be mediated by a change in cell adhesion properties of migrating neurons through the Crk/CrkL-C3G-Rap1 pathway, or by the stabilization of the cytoskeleton through the Lim Kinase-Cofilin1 pathway. Reelin signaling affects the final neuronal positioning in these nuclei but does not appear to affect their fate or their projections to target areas. Whether the aberrant position of these neurons alters their afferent connections or their functional output has not been examined.

Further studies will be necessary to elaborate the precise function of Reelin signaling in the migration of neurons in the ventral brain stem and spinal cord and to uncover whether additional ventral structures, including neuronal clusters in the forebrain, are affected in the absence of Reelin signaling. Moreover, at least some of the brainstem and spinal cord nuclei that respond to Reelin during development maintain expression of Dab1 and the Reelin receptors into adulthood, raising the question whether Reelin signaling is important for the maturation, maintenance, or function of these neurons.

## Author Contributions

ARV and SB contributed to the overall concept and the writing of the review.

## Conflict of Interest Statement

The authors declare that the research was conducted in the absence of any commercial or financial relationships that could be construed as a potential conflict of interest.
